# The role of radiotherapy for patients with primary tracheal carcinoma: two case reports of tracheal carina squamous cell carcinoma and literature review

**DOI:** 10.3389/fonc.2025.1665645

**Published:** 2025-10-02

**Authors:** Xiaojing Chang, Zhesen Tian, Yalei Zhao, Xiaohui Ge, Feng Li, Yu Yang, Huizhi Liu

**Affiliations:** ^1^ Department of Radiotherapy, The Second Hospital of Hebei Medical University, Shijiazhuang, China; ^2^ Department of Anus and Intestine Surgery, The Second Hospital of Hebei Medical University, Shijiazhuang, China

**Keywords:** primary tracheal carcinoma, radiotherapy, radiotherapy dose, chemotherapy, surgery

## Abstract

Primary tracheal carcinoma is a rare malignant tumor; its optimal treatment strategy is not yet formally proven. Surgery, especially complete surgical excision, is often considered as the first-line treatment, and chemotherapy has been reported ineffective. There is a growing body of evidence suggesting that radiotherapy may offer better local control and improve survival outcomes for cases of incomplete excision, questionable surgical margins, or unresectable lesions. However, the effect, total dose, and fraction dose of radiotherapy remain controversial. We report two cases of primary tracheal squamous cell carcinoma at the tracheal carina: a 74-year-old man with an unresectable tracheal carcinoma at the carina and a 55-year-old woman with a tracheal tumor. Both of them were treated with radical radiotherapy, demonstrating satisfactory local control. Finally, we review the current progress of radiotherapy in primary tracheal carcinoma.

## Introduction

Primary tracheal carcinoma (PTC) is a rare malignant tumor, accounting for <0.5% of all malignant tumors. Data from MD Anderson Cancer Center showed that only 74 patients have been diagnosed with primary cancers of the trachea from 1945 to June 2004, while over this 60-year period, approximately 600,000 new patients have been registered (1). Squamous cell carcinoma (SCC) and adenoid cystic carcinoma (ACC) are the most frequent histologic types (2). SCC is the most frequent histologic type in western countries (56.2% vs. 21.3%), while ACC is most common in China (50.7% vs. 30.4%) and United States (48% vs. 28%) (3–6). SCC patients appear to have poorer survival, with a 25% 5-year overall survival (OS) rate compared to those diagnosed with ACC (3, 7).

The most common symptom is dyspnea, hemoptysis, and cough. To date, surgery, especially complete surgical excision, is considered as the first-line treatment in localized PTC and shows benefit for long-term survival (8, 9). Chemotherapy was reported ineffective, but for patients with recurrent or metastatic disease, targeted and immune treatment seem to have good perspectives (10). Radiotherapy (RT) was often recommended for cases with incomplete excision, with the surgical margin being questionable, or with unresectable lesions, which could enable better local control to be achieved, but the effect, total dose, and fractionation schedules remain controversial (11, 12). Here we reported two cases of primary tracheal squamous cell carcinoma at the tracheal carina, who received radical RT that demonstrated satisfactory local control, and we reviewed the current progress of radiotherapy in primary tracheal carcinoma.

## Case presentation

### Case 1

**Figure 1:**
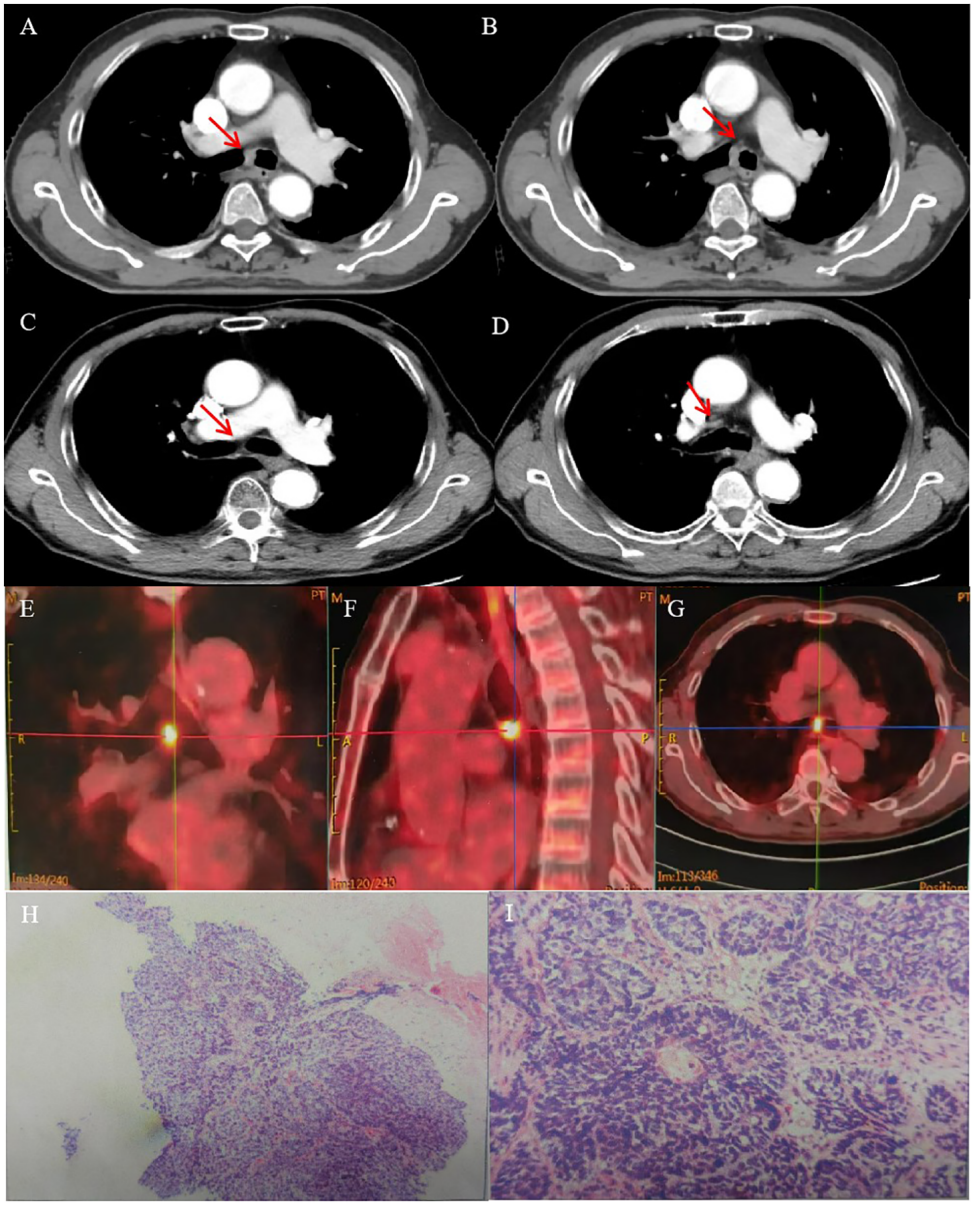
CT images and RT target of case 1 patient. **(A, B)** Computed tomography (CT) revealed a tracheal lesion at the carina. **(C, D)** The lesion disappeared 2 months after RT. **(E–G)** PET/CT revealed the thickness of the trachea of carina with a maximum diameter of 11 mm and showing strong 18F-fluorodeoxyglucose (18-FDG) uptake (SUVmax 10.6). **(H, I)** Photomicrograph of case 1 patient with primary tracheal carcinoma. The neoplasm showed the characteristic features of PTC. **(A)** ×100. **(B)** ×400.

A 74-year-old man presented with a 2-month history of intermittent coughing, expectoration, and blood-tinged sputum, without fever, dyspnea, and thoracalgia. His past medical history was characterized by a kidney stone treated with extracorporeal lithotripsy and arrhythmia (sinus arrhythmia has persisted for over 50 years without oral medication treatment). An enhanced chest computed tomography (CT) showed an opacity at the tracheal carina (**Figures 1A, B**). To verify the nature of the lesion, fiberoptic bronchoscope examination and histopathologic biopsy were performed at a local hospital. The fiberoptic bronchoscope was used to verify the lesion, and the pathological diagnosis of the biopsy was squamous cell carcinoma with focal neuroendocrine (NE) marker expression (**Figures 1H, I**). Immunohistochemistry showed the following results: CD56 (+), CgA (-), CK (+), CK5/6 (+), CK7 (+), ki-67 (+, 50%), P40 (+), Syn (–), TTF-1 (–), and NapsinA (–). Then, the patient visited our hospital, and a positron emission tomography (PET)/CT scan revealed the thickness of the tracheal carina, with a maximum diameter of 11 mm and showing a strong 18F-fluorodeoxyglucose (18-FDG) uptake (SUVmax 10.6) without distant metastasis (**Figures 1E–G**). In terms of laboratory examination, the results of routine blood and urine examinations were normal, as were those of the biochemistry examination. Based on the results above, the patient was diagnosed with tracheal SCC. The American Joint Committee on Cancer (AJCC) does not have TNM stage definitions for tracheal tumors; however, the stage was cT4N0M0, stage IIIA according to AJCC for lung cancer staging or cT1N0M0, stage I according to the classification proposed by Bhattacharyya (13).

**Figure 2:**
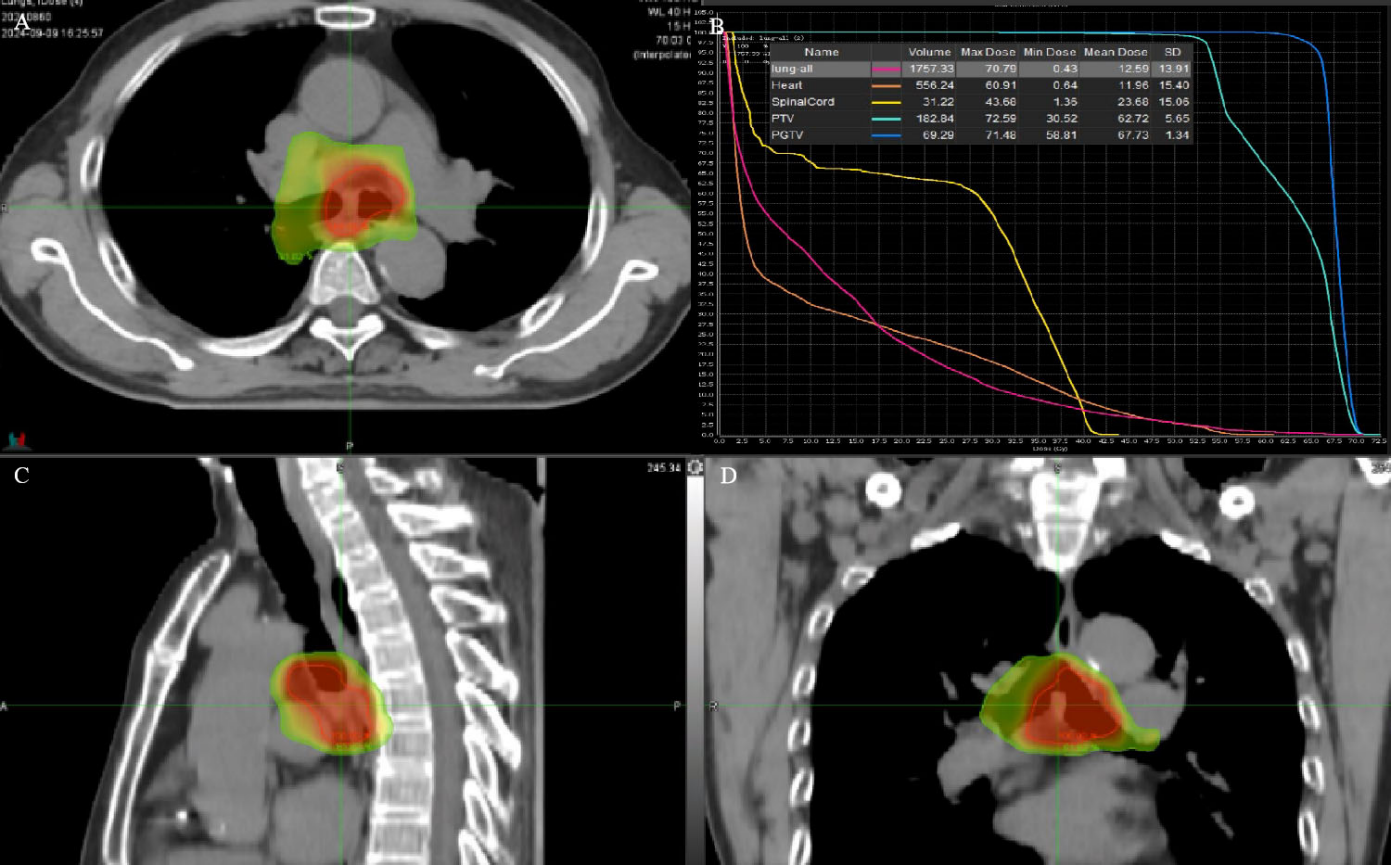
**(A, C, D)** RT target; area of red line: 66 Gy; and area of yellow line: 54 Gy. **(B)** DVH of case 1 patient.

The patient refused an operation due to his advanced age. He received intensity-modulated conformal radiation therapy (IMRT) of TOMOtherapy (USA), the target volume of which was described as follows: according to contrast-enhanced CT and PET/CT images to delineate the target, the gross tumor volume (GTV) comprised a gross tumor, the clinical target volume (CTV) had an area of 2 cm above and below the tumor, and CTV was enlarged 5 mm circumferentially to form the planning target volume (PTV). GTV was also enlarged 5 mm circumferentially to form the PGTV. The dose of PGTV was 66 Gy in 33 fractions of 2 Gy, while that of PTV was 54 Gy/30f (**Figures 2A–D**).

The patient had no obvious side effects during RT, only mild nausea and fatigue. He recovered well after RT without chemotherapy. At 2 months later, the CT result showed that the trachea was unobstructed and that there was no recurrence (**Figures 1C, D**). At 6 months after RT, he reported no blood-tinged sputum, and his body movement had returned to normal. The authors confirm that written informed consent was provided by the patient for the publication of this case report and the inclusion of accompanying images.

### Case 2

**Figure 3:**
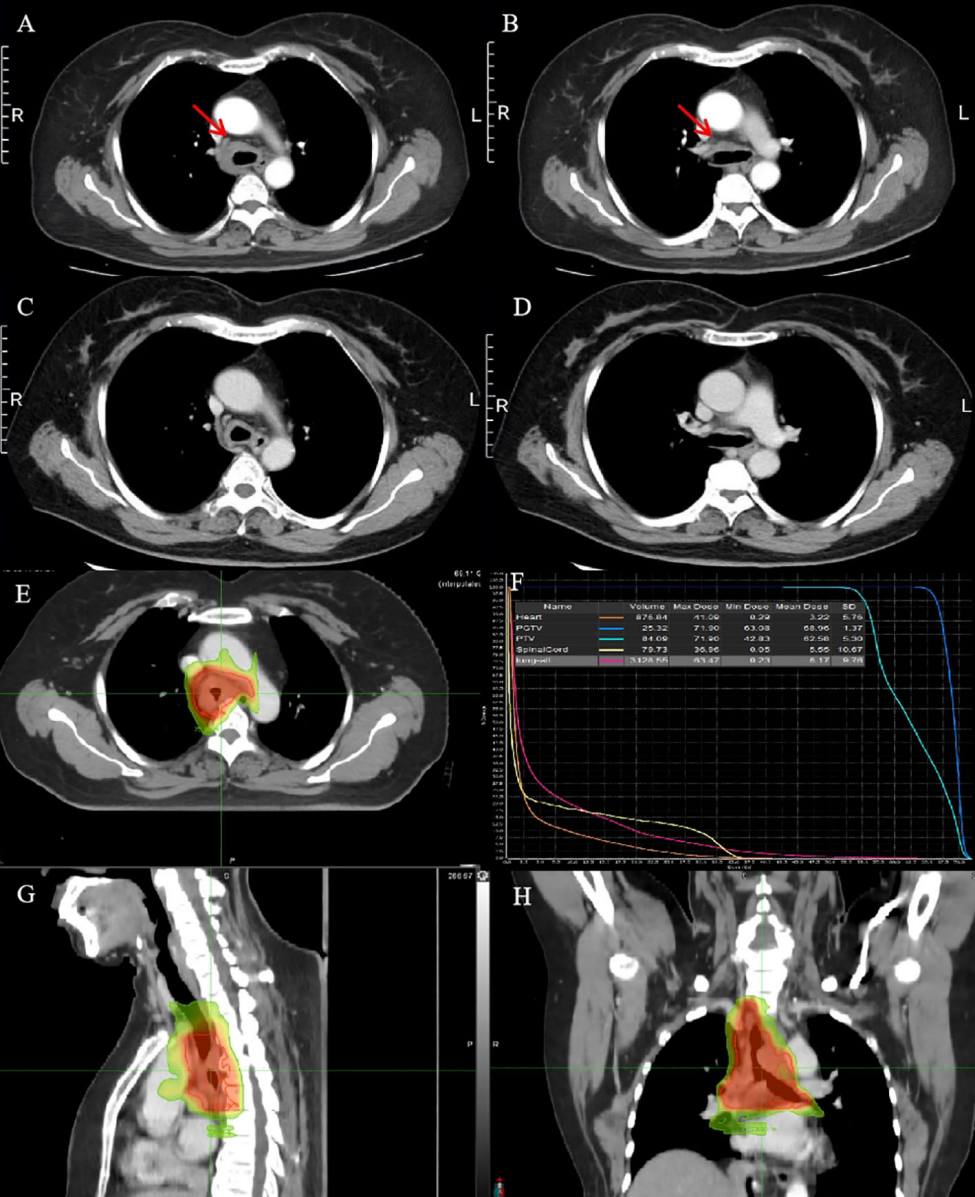
CT images and RT target of case 2 patient. **(A, B)** Computed tomography (CT) revealed a tracheal lesion. **(C, D)** The lesion was reduced at 1 month after RT. **(E–H)** RT target and DVH of case 2 patient; area of red line: 66 Gy; and area of yellow line: 54 Gy.

**Figure 4:**
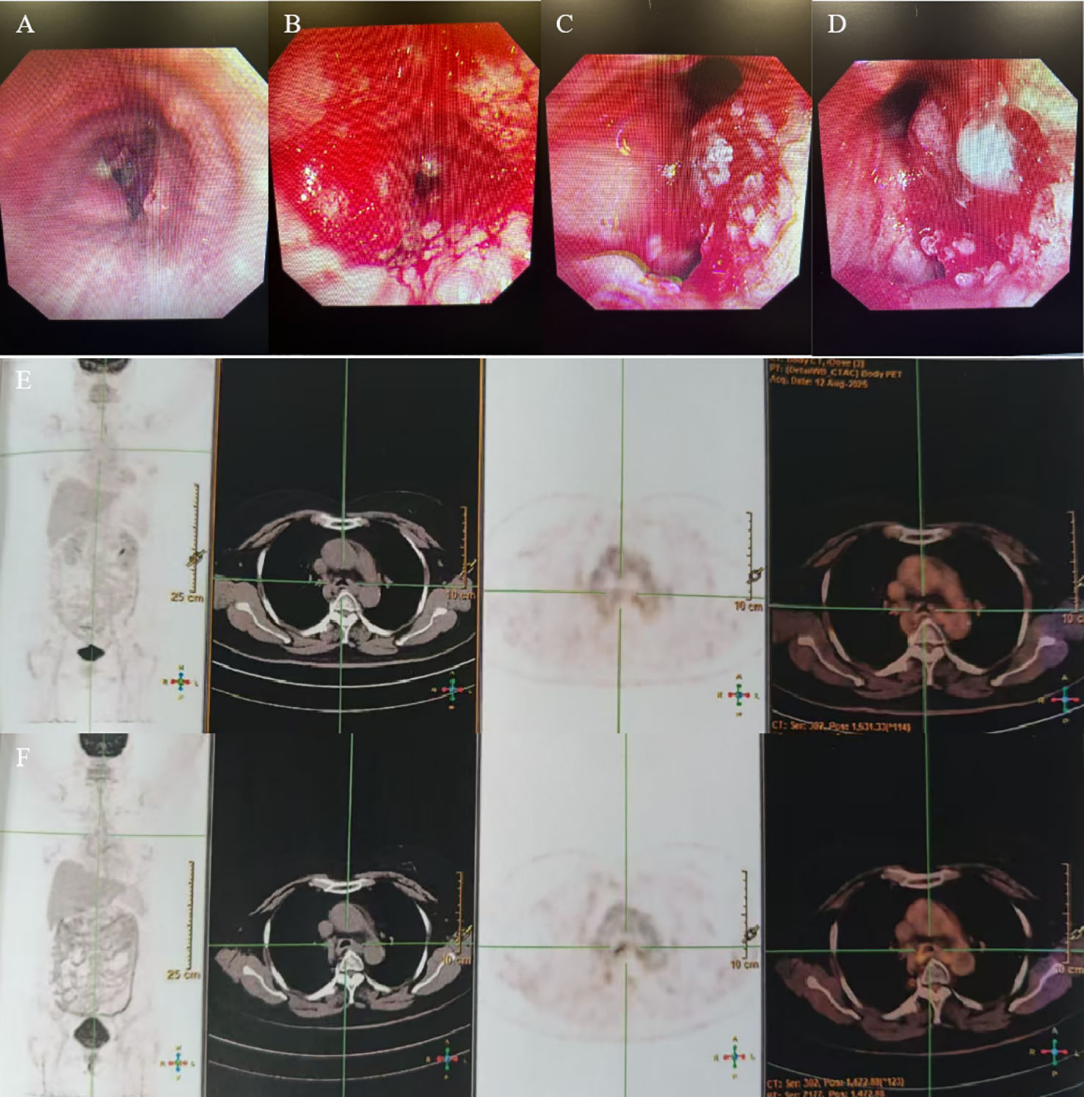
**(A–D)** Fiberoptic bronchoscopy showed a trachea lesion situated 3 cm above the carina, and the lumen appeared notably narrow. **(E, F)** PET/CT revealed the thickness of the tracheal carina, showing no 18-FDG uptake and without distant metastasis.

A 53-year-old woman presented with a history of more than 1 year of cough, coughing up phlegm, and shortness of breath for half a year, which worsened for half a month prior to her current presentation. Notably, she did not experience fever, thoracalgia, or any other relevant symptoms during that time. She denied any history of other diseases. The chest CT revealed the marked thickness of the lower segment and tracheal carina, leading to a locally narrowed trachea (**Figures 3A, B**). She had been hospitalized at local hospitals and received pharmaceutical treatment aimed at relieving her symptoms before her admission to our hospital. Regrettably, her shortness of breath did not yield with a significant relief. Subsequently, she was referred to our hospital for further medical assessment. After a whole-body examination was completed, there was no distant metastases found. The preliminary diagnosis pointed to an intratracheal mass. A fiberoptic bronchoscope verified a tracheal lesion situated 3 cm above the carina, and the lumen appeared notably narrow (**Figures 4A–D**). The pathological diagnosis of the biopsy was squamous epithelial high-grade intraepithelial lesion, with a tendency to be cancerous at the focal lesion. The tumor markers showed the following result: squamous cell carcinoma antigen at 2.74 ng/ml. The remaining examinations, as well as the laboratory examination, including routine blood, urine examinations, and those of the biochemistry examination, showed no abnormalities. The patient was diagnosed with tracheal SCC (cT4N1M0, stage IIIA) according to AJCC for lung cancer staging, but according to the classification proposed by Bhattacharyya, the stage was cT2N1M0, stage IIB. The surgery was difficult and risky, and radiation therapy was recommended. The patient received IMRT of true beam (Varian, USA). The target volume and radiation dose were as described in case 1 above combined with four cycles of chemotherapy of carboplatin plus paclitaxel (**Figures 3E–H**).

The patient had no obvious side effects during RT and recovered well. The CT result showed that the trachea lesion was markedly reduced at 1 month after RT (**Figures 3C, D**). She received four cycles of chemotherapy. At 6 months after RT, the chest CT still showed the thickness of the original tracheal lesion site, so a PET/CT was performed, which revealed the thickness of the tracheal carina and showed no 18-FDG uptake as well as without distant metastasis at nearly 9 months after RT (**Figures 4E, F**). Since then, she had undergone regular follow-up examinations every 3 months, and no symptom was reported. Her body movement though had returned to normal during the nearly 1-year telephonic follow-up. The patient provided written informed consent for the publication of the case details and the inclusion of accompanying images.

## Discussion

Distinct from primary lung carcinoma which is also known as primary bronchial lung carcinoma, tracheal tumor is mainly located in the trachea. The two presented cases showed that the lesions are both located in the trachea: case 1 showed no invading in the left and right bronchi, and case 2 showed the trachea lesion situated 3 cm above the carina; and the combined results of imaging and bronchoscopy support the diagnosis of tracheal malignant mass.

To date, there is no standard TNM staging and treatment guidelines for PTC. The classification proposed by Bhattacharyya seems more suitable compared with the AJCC staging of primary bronchial lung carcinoma (13). For decades, surgical excision combined with airway reconstruction had been considered as the preferred treatment, especially for those with severe life-threatening airway stenosis, and the patients had a good result from surgery (14). A case report described a 74-year-old female patient with PTC who had undergone surgery alone. The pathology results showed that the tumor invaded the adventitia of the trachea and bilateral thyroid. The patient recovered well, and no recurrence was found at 8 months after the operation (15). Another report showed a PTC that was excised using interventional bronchoscopy including an electric snare, electrotomy, cordectomy, and an argon knife. The patient achieved complete recovery for 2 years without any radiotherapy or chemotherapy (16). A retrospective study enrolled 270 patients (135 patients with SCC and 135 with ACC) with PTC. The 5-year OS was 39.1% and 52.4% for resected SCC and ACC patients, respectively. However, it was only 7.3% and 33.3% for unresectable SCC and ACC patients, respectively (8). Despite improved survival, the prognosis for patients with PTC is still poor; the 5-year overall survival (OS) was 31.7% (17). It means that adjuvant therapy combined with resection may delay the patients’ relapse and metastasis.

To date, chemotherapy was reported ineffective, but an increasing number of studies showed that RT was considered as an independent prognostic factor for cases with incomplete excision, with the surgical margin being questionable, or with unresectable lesions. RT could achieve better local control, delay the patients’ relapse and metastasis, and prolong the survival time (**Table 1**), but postoperative chemotherapy following RT in patients with incomplete resection did not seem to show an additional survival benefit (23, 32). Due to the lack of large randomized clinical trials, the benefit of radiotherapy is not clinically proven, and the total dose and fractionation schedules remain unestablished. Zheng Z et al. (33) analyzed the impact of RT on PTC patients using the data from SEER database. The results showed that the OS was better in patients who received RT compared to those who did not receive RT (median OS: 12 vs. 4 months). Unfortunately, the RT doses were not analyzed and recommended. Yang CJ et al. (34) and Yusuf M et al. (35) retrospectively analyzed the survival value of RT on PTC patients with positive margins and found no significant OS benefit between patients who did or did not receive postoperative RT. Unfortunately, the authors did not give details on the radiotherapy doses.

**Table 1:** RT dose and survival using traditional RT in PTC.

Year	Authors	Study type	Tumor type	Number of cases	RT type (PORT/DRT)	Radiotherapy	Effect
1998	Mornex F et al. (18)	Case series	SCC/ACC	DRT: 84 HDR RT: 5	DRT HDR RT	Mean dose: 56 Gy (range: 30 to 70 Gy, 2 Gy/f) HDR RT: 5–7 Gy/2–3f	DRT: ORR 89% (CR 51%; PR 38%); >56 Gy: CR 65%, 1-, 2-, 5-year OS was 64%, 35%, 12%; <56 Gy: CR 28%, 1-, 2-, 5-year OS was 23%, 10%, 5%
2010	Hetnał M et al. (19)	Case series	SCC/ACC	50 (SCC: 24)	DRT/palliative RT	DRT: 64 Gy Palliative RT: 35 Gy	PR: 73%, median OS: 8.7 months;
2010	Ly V et al. (20)	Case report	SCC	1	PORT	66.6 Gy/37f	CR was shown by PET-CT 16 months after RT
2010	Abbate G (21)	Case report	SCC	1	Palliative RT	50.4 Gy/30f, boosts to 61.2 Gy	Recurrence about 4 months after RT, and the patient died 1 year after diagnosis
2012	Bonner Millar LP et al. (22)	Case report	ACC	2	DRT	64 Gy/30f	Case 1: no recurrence during 5-year follow-up; case 2: no recurrence 11 months after treatment
2015	Chen F et al. (23)	Case series	ACC	DRT: 4 PORT: 24 (incomplete resection: 37; PORT: 24 NO PORT: 13	PORT/DRT	PORT: 40–60 Gy DRT: 60–66 Gy (2.0 Gy/f) A boost dose was provided for surgical Margin	DRT; 4 patients had no disease progression during 68 months of follow-up; PORT: median DFS and OS was 92 and 125 months; NO PORT: median DFS and OS was 62 and 78 months
2016	Agrawal V et al. (24)	Case report	SCC	2	DRT	Case 1: 60 Gy/30f Case 2: 66 Gy/33f	Case 1: recurrence at 12 months with renal metastasis Case 2: CR, no evidence of disease at 14 months
2017	Je HU et al. (25)	Case series	ACC	PORT: 13 DRT: 9	PORT/DRT	DRT: 60–66 Gy PORT: 50.4 Gy (a boost dose of 15 Gy or 21 Gy with 5–7 Gy per fraction for surgical margin)	DRT: ORR 77.8% 5-year LRPFS: PORT: 100%; DRT: 66.7% 5-year OS: PORT: 92.3%; DRT: 66.7% 10-year OS: PORT: 76.9%; DRT: 22.2%
2018	Levy A et al. (26)	Case series	ACC	PORT: 22 DRT: 9	PORT/DRT	PORT: 45–66 Gy DRT: 66–70 Gy Mean dose: 62 Gy (42–70 Gy, 2 Gy/f)	5-year local relapse rate: DRT: 10% PORT: 0%
2019	Spinelli G et al. (27)	Case report	ACC	1	PORT	70 Gy/35f (2.0 Gy/f)	CR was shown by PET-CT after 6 and 12 months of follow-up
2021	Zeng R et al. (28)	Case series	SCC/ACC	PORT: 13 DRT: 18	PORT/DRT	PORT: 50–54 Gy DRT: 60–70 Gy (1.8–2 Gy/f)	DRT: ORR 80.7% (CR 44.4%; PR 55.6%) 5-year LRPFS rate: PORT: 91.7%; DRT: 50.1%
2022	Dracham C et al. (29)	Case series	ACC	PORT: 3 DRT: 12	PORT/DRT	PORT: median 50 Gy DRT: 67.8 (54–90 Gy) (7 cases with 1.8–2 Gy/f, 5 with 4 Gy/f)	DRT: ≧̸66 Gy had better survival (5-year LRFS: 75% vs. 6.7%)
2024	Krishnasamy S et al. (30)	Case report	ACC	1	PORT	80 Gy/40f (2.0 Gy/f)	No disease recurrence at 18 months post-surgery
2024	Lee JH et al. (31)	Case series	ACC	PORT: 22 DRT: 26	PORT/DRT	PORT: 57–74 Gy High-dose group: 60–74 Low-dose group: 57–60 DRT: 45–74 Gy High-dose group: 60–74 Low-dose group: 45–66	DRT: ORR 100% (CR 3.8%; PR 76.9%) 5-year OS: PORT: 100%; DRT: 69.7%; 5-year FFLP: PORT: 90.5%; DRT: 76.7%

PORT, post-operative radiotherapy; DRT, definitive radiotherapy; SCC, squamous cell carcinoma; ACC, adenoid cystic carcinoma; CR, complete remission; PR, partial remission; f, fraction; LRPFS, loco-regional progression-free survival; HDR RT, endoluminal high-dose-rate brachytherapy; FFLP, freedom from local progression.

A study showed that the radiation dose may affect local control and the patients’ OS. The 5-year OS dropped from 12% for patients receiving doses greater than 56 Gy to 5% for lower doses, and it is recommended to administer greater than 60 Gy for primary irradiation in PTC (18). Abbate G et al. (21) reported a 54-year-old male patient diagnosed with primary SCC, with a voluminous tracheal mass of approximately 6 to 7 cm in length, and was treated with palliative RT with a total dose 50.4 Gy. He was given boosts on the trachea up to 61.2 Gy, but recurrence was observed at approximately 4 months after RT. Hetnał M et al. (19) analyzed the role of RT in 50 patients with PTC (24 cases with SCC). The median dose was 64 Gy (range, 56–70 Gy) with radical RT, and 73% of the patients showed PR. Ly V et al. (20) reported a 75-year-old man with primary tracheal SCC who received a total of 66.6 Gy of RT at 1.8 Gy per fraction after electrocautery, argon photocoagulation, and cryotherapy. He achieved CR, and there was no recurrence during the 16-month follow-up period. In a retrospective study, 18 PTC patients received definitive RT. The sub-group univariate analysis indicated that the 5-year progression-free survival (PFS) was better for those who received at least 68 Gy of radiation (28). Agrawal V et al. (24) reported two cases with SCC of PTC; one was treated with 60 Gy RT in 30 fractions, and the other received a total of 66 Gy in 33 fractions, with both achieving complete response (CR). Therefore, 66 Gy of radical RT was recommended for primary SCC.

For ACC, due to its being less radiosensitive, higher doses may be needed. Levy A et al. (26) found that a dose of radiotherapy <60 Gy was associated with a decreased PFS for tracheal ACC. An earlier case report recommended a dose of 70–80 Gy with acceptable toxicities for tracheal ACC patients (22, 27). Krishnasamy S et al. (30) reported a 27-year-old lady who underwent IMRT to the surgical positive margins at 64 Gy/30f. There was no recurrence after 18 months of follow-up. A newly retrospective study evaluated the efficacy of dose-escalated RT for primary tracheobronchial ACC by dividing 48 patients into low (<70.0 Gy, range: 56.3–69.3 Gy) or high (≥70.0 Gy range: 70.0–82.5 Gy) RT dose groups. The results showed that the 5-year OS were 88.2% and 100% in the postoperative RT group (*p* = 0.230) and 66.7% and 79.0% in the definitive RT group, respectively (*p* = 0.022). Thus, a radiation of ≥70.0 Gy could be considered a primary treatment option for patients with unresectable lesions for several reasons (31). Je HU et al. (25) analyzed the effect of adjuvant or definitive RT for primary tracheal ACC. The dose was 60–66 Gy of conventional fractionation for definitive RT. Dracham C et al. (29) reported 12 patients who received definitive RT with 54–90 Gy (median dose of 67.8 Gy), and they found that patients receiving a higher RT dose (≥66 Gy) had significantly better survival outcomes. Thus, ≥66 Gy of radical RT was recommended for tracheal ACC.

For the fraction dose, Je HU et al. (25) reported the range from 1.8 to 2.2 Gy. In the study of Yang Y et al., the fraction dose was 2.0–2.14 Gy (36). Another retrospective study showed that it was varied from 1.6 to 3 Gy (median, 2 Gy), with the total dose ranging from 42.5 to 82.6 Gy (median, 66.0 Gy) in the radical RT group (2). Hetnał M et al. (19) reported that the dose per fraction ranged from 1.8 to 2 Gy in radical RT, while it was 3 to 4 Gy in palliative RT. In our center, the two patients both received definitive RT with 66 Gy in 33 fractions of 2 Gy and achieved better local control; the two patients both showed CR. Therefore, we suggest 66.0 Gy for definitive RT as preferable for SCC, but a higher dose for locally advanced ACC patients with acceptable toxicities. It is worth noting that the pathological diagnosis of case 1 was SCC with focal NE marker expression. As we all know, NE markers are important markers to differentiate and diagnose NE tumors, which means relatively high sensitivity with RT and chemotherapy, especially poorly differentiated NE cancers, although exhibiting aggressive characteristics and a higher recurrence rate, such as small cell lung cancer. The study showed that conventional non-small lung cancer (NSCLC), such as adenocarcinoma and SCC, does not exhibit a NE morphology but does express NE marker(s), named NSCLC with NE differentiation (37, 38). For case 1, it was SCC with NE differentiation, which could explain why the effect of RT alone was better and CR was achieved, although the stage was cT1N0M0, stage I according to the classification proposed by Bhattacharyya. Due to his advanced age, the patient did not receive chemotherapy. Future work is needed to understand the role of NE differentiation in PTC, which may help promote a more accurate diagnosis and develop specific treatment strategies.

For target delineation, GTV included the gross tumor or postoperative tumor bed along with positive lymph nodes. The CTV of postoperative RT had an area of 3 cm above and below the surgical anastomosis including the tumor bed and the draining area of the pathologically positive lymph nodes, while the CTV of definitive RT expanded 3 cm above and below the tumor with the clinically positive nodal regions based on contrast-enhanced CT scans (25, 30). In the report of Dracham C et al. (29), CTV was generated using a 1-cm longitudinal and 1-cm radial margin. In our center, the CTV of definitive RT for these two patients was also the area 2 cm above and below the tumor with the clinically positive nodal regions. PTV was generated with 5 mm margins around the CTV.

For RT techniques, in a retrospective study of 133 cases, 66 patients with positive surgical margins were divided into non-IMRT (two-dimensional RT, 2D-RT, mainly) and IMRT groups. The results showed that the OS of non-IMRT patients showed no significant improvement in comparison with the no-RT patients (the 5-year OS was 70.2% vs. 77%, and the 10-year OS was 45.4% vs. 47.9%), whereas the 5-year (94.7%) and 10-year OS (82.9%) of the adjuvant IMRT group were significantly better than the no-RT group and the non-IMRT group (22). Modern advanced RT techniques such as proton and carbon ion beams have physical advantages and can provide specific better dose distribution and better sparing of normal tissue. In a study that enrolled 18 patients with primary tracheobronchial adenoid cystic carcinoma who received doses of CIRT at 66–72.6 GyE/22–23 fractions, the overall response rate (ORR) was 88.2%, and the 2-year OS and PFS were 100% and 61.4%, respectively. However, considering the expensive fees, it is not suitable for a wide range of clinical applications (39). Nakamura M et al. (40) reported two cases of PTC of the trachea that received proton beam therapy with 74-Gy dose in 37 fractions; both of them achieved long-term survival. Furthermore, brachytherapy may be used for tracheal tumors, even as a boost for external beam irradiation. Carvalho Hde A (41) reported four patients with nonresected primary tracheal tumors who received brachytherapy—two cases of SCC, one case of recurrent ACC, and one case with recurrent plasmacytoma—in three or four fractions of 7.5 Gy, calculated at a depth of 1 cm. All patients presented complete local response at the time of the first bronchoscopic evaluation. Nguyen NT (42) reported eight patients who received brachytherapy alone with 5–7 Gy/fraction, using one to three fractions. The patients experienced symptomatic improvement and good local response, but large randomized clinical trials are needed to prove the role of brachytherapy in PTC.

Chemotherapy was reported ineffective. In recent years, immune checkpoint inhibitors (ICIs), including anti-PD-1, anti-PD-L1, and anti-cytotoxic T lymphocyte antigen 4, have emerged as promising therapeutic agents in those patients with recurrent or metastatic disease. Immunotherapy also seems to have good perspectives in PTC (10). Mikami E et al. (43) and Nakatani Yu et al. (44) both reported one PTC patient who received definitive concurrent chemoradiotherapy followed by immunotherapy (durvalumab) and who achieved successful treatment and prognosis. Immunotherapy may be a promising treatment option for unresectable or recurrent and metastatic PTC patients.

## Conclusions

In conclusion, PTC is a rare malignant tumor. For incomplete excision, a positive surgical margin, or unresectable lesions, RT could achieve better local control, delay the patients’ relapse and metastasis, and prolong the survival time. A dose of 66.0 Gy for definitive RT using IMRT or VMAT was preferred for SCC and a higher dose for ACC (such as ≥66.0 Gy) patients with acceptable toxicities. Other RT techniques such as proton and carbon ion beams are not suitable for a wide range of clinical applications. Large randomized clinical trials are urgently needed to prove and recommend the RT dose for patients with PTC. Furthermore, target and immunotherapy may be a promising treatment option for unresectable lesions or recurrent and metastatic PTC patients.

## Data Availability

The original contributions presented in the study are included in the article/Supplementary Material. Further inquiries can be directed to the corresponding author.
